# Formation of inclusion complex of enrofloxacin with 2-hydroxypropyl-*β*-cyclodextrin

**DOI:** 10.1080/10717544.2020.1724210

**Published:** 2020-02-24

**Authors:** Yili Ding, Yuchang Pang, Chamakura V. N. S. Vara Prasad, Bingyun Wang

**Affiliations:** a Life Science Department, Foshan University, Foshan, P. R. China;; b Das Pharma, Kakinada, India

**Keywords:** Enrofloxacin, 2-hydroxypropyl-*β*-cyclodextrin, inclusion complex, preparation, pharmaceutical properties

## Abstract

Enrofloxacin, a third-generation fluoroquinolone, is a broad-spectrum antimicrobial drug against a lot of veterinary bacterial diseases. However, bactericidal activity of enrofloxacin is concentration-dependent and its poor aqueous solubility and bitter taste limit its development and application. Meanwhile, 2-hydroxypropyl-*β*-cyclodextrin (HP-β-CD), a widely used cyclodextrin analog, is a safe and an effective drug carrier. It forms inclusion complexes with its drug substrates and improves their physiochemical and pharmacokinetic properties. Enrofloxacin was also found to form a stable inclusion complex with HP-β-CD and different research groups have shown improved solubility for enrofloxacin by 32.5%, 9.25 and 165-fold. Our own efforts in this direction resulted in manifold improvement (916-fold) in its solubility compared to the previous studies. It was further shown that pharmaceutical properties, absorption and bioavailability, of enrofloxacin have also been significantly improved by complexation with HP-β-CD.

## Introduction

**Figure 1.**
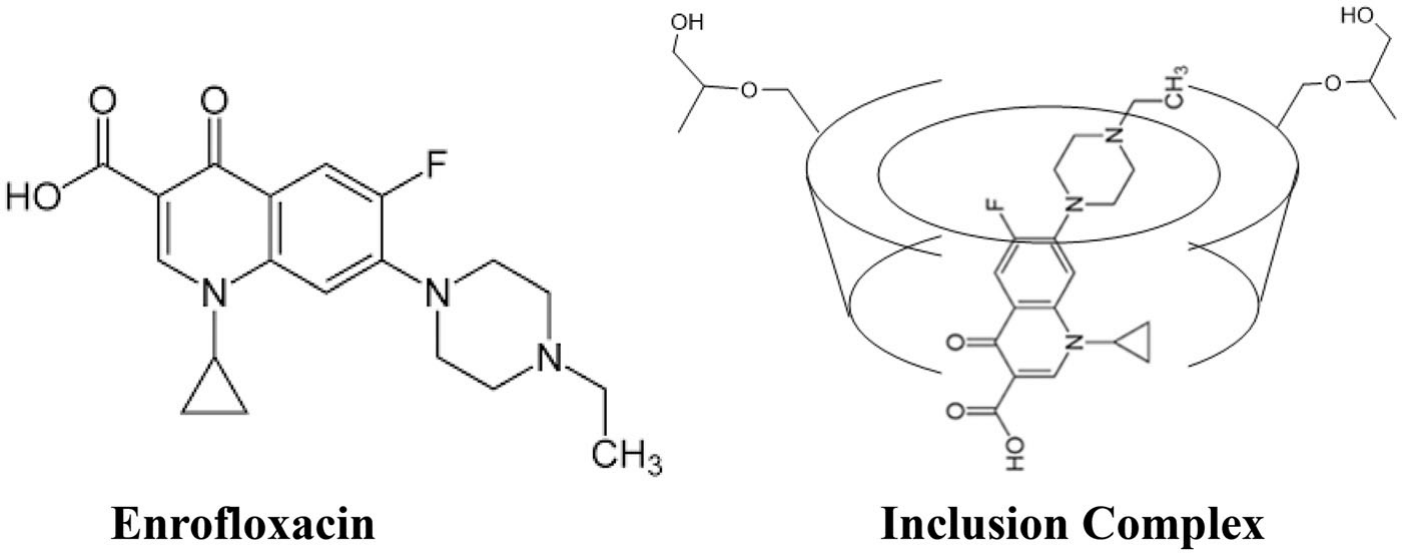
Structures of enrofloxacin and enrofloxacin–HP-β-CD inclusion complex.

Enrofloxacin (Figure 1), or 1-cyclopropyl-6-fluoro-7-(4-ethyl-1-piperazinyl)-1,4-dihydro-4-oxo-3-quinoline-carboxylic acid, belongs to fluoroquinolone family which is a subfamily of quinolone (Hooper & Wolfson, 1985) and is the third generation fluoroquinolone antimicrobial drug with broad and strong anti-bactericidal activity against a lot of bacterial diseases (Sarkozy, 2001). Its high lipophilic property, carboxylic acid, and tertiary amine functional groups contribute to its amphoteric properties (Vancutsem et al., 1990). It can be used to treat specific infections and against a broad spectrum of Gram-negative and Gram-positive bacteria in both stationary and growth phases of bacterial replication (Scheer, 1987). Its wide *in vivo* distribution, unique antimicrobial effect, high bioavailability, less toxicity, and side effects, make it one of the most commonly used antibiotics for treatment of various animal infectious diseases, and a desirable antibiotic choice for difficult-to-treat infections, particularly those that need long-term antibiotic treatment (Divers et al., 2008; Ebert et al., 2011; Reyes-Herrera et al., 2011; Jerjomiceva et al., 2014; Lin et al., 2014; Rico et al., 2014; Andrieu et al., 2015; Nguyen Dang Giang et al., 2015; Phillips et al., 2015; Piras et al., 2015; Carrascosa et al., 2017; Foster et al., 2017; Roth et al., 2017; Strzępa et al., 2017; Zhu et al., 2017; Rico et al., 2018). The bactericidal activity of enrofloxacin is concentration-dependent, with susceptible bacterial cell death occurring within 20–30 minutes of exposure. However, the poor aqueous solubility and bitter taste of enrofloxacin limit its development and application (Baluja et al., 2008; Seedher & Agarwal, 2009).

The problem of the low solubility of many drugs has been overcome by complexation of the active principle ingredient with *α*-, *β*-, or *γ*-cyclodextrins, and 2-hydroxypropyl-*β*-cyclodextrin (HP-β-CD) as well. HP-β-CD, is the most widely used modified cyclodextrin, has excellent inclusion properties for many compounds, is less toxic, safe, and an effective drug carrier (Gould & Scott, 2005; Misiuk & Zalewska, 2009; Folch Cano et al., 2014; Srivalli & Mishra, 2016; Carneiro et al., 2019). Previously, HP-β-CD was used to form the inclusion complex with enrofloxacin to increase its aqueous solubility and stability. In this regard, Zadra reported 32.5% increase in the solubility of enrofloxacin through the formation of inclusion complex with HP-β-CD (Zadra et al., 2009). Furthermore, de Moraes reported the formation of inclusion complex of enrofloxacin with HP-β-CD, which resulted in a 9.25-fold increase in enrofloxacin solubility in comparison to that of in the absence of HP-β-CD (Calsavara et al., 2012). Further, Wang et al. reported the preparation of the inclusion compound of enrofloxacin with HP-β-CD by using the stirring method, and the solubility of the inclusion complex has improved by 165-fold over that of enrofloxacin alone (Wang et al., 2012) without the aid of HP-β-CD.

In continuation of our interest in studies (Ding et al., 2018) with cyclodextrin molecules, we have been interested to improve the solubility of enrofloxacin using HP-β-CD inclusion complexes. To our surprise and dismay, the literature procedures to prepare the inclusion complex of enrofloxacin and HP-β-CD produced inconsistent results with regard to enrofloxacin’s water solubility, which prompted us to investigate the literature procedures in detail. After careful repetition of each reported experiment with subtle modifications, we achieved even much better results. Thus, it is hypothesized that smaller changes in the reaction conditions for the formation of the inclusion complex of enrofloxacin with HP-β-CD might make big difference for enrofloxacin’s water solubility, which will have a direct bearing on its *in vivo* pharmacokinetic properties such as absorption and bioavailability. In this communication, we like to report the preparation and characterization of the inclusion complex of enrofloxacin with HP-β-CD in detail, and its *in vivo* pharmacokinetic evaluation.

## Enrofloxacin inclusion complex

### Experimental

#### Materials and instruments

Enrofloxacin with 99.9% purity was purchased from China Veterinary Pharmaceutical Supervision Bureau, HP-β-CD, anhydrous ethanol, and acetic acid with analytical purity were obtained from Tianjin Guangcheng Chemical Reagent Company (Tianjin, China). ^1^H NMR spectra were recorded by Bruker spectrometer (Billerica, MA, USA) operating at 400 MHz using D_2_O and DMSO-D_6_ as lock solvents; the FTIR spectra were recorded on IR200 Fourier transform infrared spectrometer (Jiangsu Jintan Shuangjie); high-performance liquid chromatographic analysis was performed over Shimadzu LC-20A high-performance liquid chromatography instrument; the scanning electron microscopic images were recorded on a micro-surface morphology (Tecnai G2 Spirit Biotwin, FEI, Hillsboro, OR, USA) equipment; the dissolution testing was performed in RC-3 Dissolution tester (Tianjin Xintianguang Analytical Instrument Technology, Tianjin, China); 24 SPF rats were obtained from Guangdong Medical Laboratory Animal Center (Foshan City, China); the blank rat plasma was obtained from Guangzhou Rui-Te Co. Ltd. (Guangzhou, China).

#### General Procedure for preparation of the inclusion complex of enrofloxacin and HP-β-CD

HP-β-CD was dissolved into distilled water at room temperature, the solution of enrofloxacin in acetic acid was added slowly, and the solution was stirred at a certain speed at 55 °C for few hours. The mixture was cooled to room temperature and put into a refrigerator at 4 °C for 24 hours. The solid was collected through filtration, washed with small amount of ethanol, and dried at 55 °C under vacuum for 24 to provide the inclusion complex as a white solid.

#### Characterization of the inclusion complex

The inclusion complex was characterized by proton NMR and FTIR spectral study. FTIR spectra of the KBr pellets of enrofloxacin, HP-β-CD, and the inclusion complex of enrofloxacin and HP-β-CD were obtained using IR200 Fourier transform infrared spectrometer (China). Data were acquired between 4000 and 400 cm^−1^. The HP-β-CD and the inclusion complex of enrofloxacin with HP-β-CD were dissolved into D_2_O; enrofloxacin was dissolved into DMSO-D_6_; their proton NMR spectra were recorded at 400 MHz at room temperature and scanned for 16 times.

#### Scanning electron microscopic images of inclusion complex

The surface morphology of enrofloxacin, HP-β-CD, the mixture of enrofloxacin and HP-β-CD, and the inclusion complex of enrofloxacin with HP-β-CD were studied using a Tecnai G2 Spirit Biotwin (FEI, Hillsboro, OR, USA) scanning electron microscope at an accelerating voltage of 10 kV. The solid sample was placed on the magnetic block, after gold spray treatment for 30 minutes; the sample was loaded onto the sample rod for scanning. The image magnification is 500 times, and the image type is secondary electronic image.

#### UV spectrometry study of enrofloxacin, HP-β-CD, and the inclusion complex

The ultraviolet spectroscopic observations of the aqueous solutions of enrofloxacin, HP-β-CD, and the inclusion complex were performed in the wavelength range of 200–400 nm by using the distilled water as a blank. It was found that enrofloxacin had the maximum absorption at 278 nm, while HP-β-CD had no absorption, thus, 278 nm was chosen as the determining wavelength in HPLC analysis.

#### Method for the determination of enrofloxacin in the inclusion compound

Enrofloxacin (10 mg) was dissolved in methanol (2 mL), and the solution was diluted to a concentration of 0.4 mg/mL by adding distilled water. From this stock solution, a series of solutions with different concentrations of enrofloxacin were obtained and analyzed by HPLC (C-18 column: 250 mm × 4.6 mm, 5 µm; mobile phase: 0.025 mol/L aqueous phosphoric acid and acetonitrile in a ratio of 83:17; wavelength in UV detector: 278 nm). Enrofloxacin methanolic aqueous solutions with different concentrations (31.25–1000 µg/mL range) were analyzed by HPLC, the absorption peak areas in HPLC traces and concentrations showed a good linear relationship, the standard regression curve equation and correlation coefficient were obtained as *Y* = 10^−8^*X*–0.0066 (*R*^2^=0.9999; *Y*: absorption peak area; *X*: concentration).

#### Determination of the content of enrofloxacin in the inclusion complex

One hundred milligrams of the inclusion complex of enrofloxacin with HP-β-CD was dissolved in 1 mL of deionized water, the solution was then diluted to the ranges of 31.25–1000 μg/mL for HPLC analysis. The concentration of enrofloxacin in inclusion complex was obtained through the regression equation.

#### Determination of solubility of enrofloxacin in water as inclusion complex

The inclusion complex was added to water (1 mL) with stirring to get its saturated aqueous solution, which was analyzed by HPLC to know the solubility of enrofloxacin in water.

#### Determination of inclusion rate and inclusion yield

The inclusion rate and yield of inclusion compound were used to evaluate the inclusion effects of inclusion complexes. The inclusion rate and yield of enrofloxacin inclusion compound could be calculated through the following formulas:

Inclusion yield (%) =[enrofloxacin inclusion complex (mg)/enrofloxacin (mg) + (HP−β−CD(mg)]×100%

Inclusion ratio (%)=[enrofloxacin in inclusion complex (mg)/enrofloxacin (mg)]×100%

#### Dissolution determination

The dissolution tests are performed over the USP Apparatus 2 (paddle) by using the degassed deionized water (900 mL) as the medium at 37 ± 0.3 °C with 100 rpm rotating speed. Enrofloxacin (100 mg), inclusion complex (containing 100 mg of enrofloxacin), and the mixture of enrofloxacin (100 mg) and HP-β-CD (430 mg) were used for the testing. The solution (5 mL) for each test was collected at 2, 5, 10, 15, 20, 30, 45, and 60 min, respectively. After each collection, 5 mL of isothermal medium was supplemented. The collected solution was filtered through 0.22 µm microporous membrane and analyzed by HPLC, each testing was repeated three times, the dissolution rate was obtained from the standard regression curve equation.

#### Pharmacokinetic studies

After one week of adaptation in the new environment, 24 SPF rats were randomly divided into two groups. After 12 hours fast and collections of blank blood samples, the rats were dosed with enrofloxacin and its inclusion complex at a single dose of 5 mg/kg of body weight respectively. The rats were resumed to normal diet with complementary foods and drinking water after the drugs were dosed for two hours. After drug administration, 2 mL of blood sample was collected at 0.25, 0.5, 0.75, 1, 2, 3, 4, 6, 8, 12, 24, 36, 48, and 72 hours respectively with a 5 mL syringe containing the heparin sodium from each rat. The blood sample was centrifugated at 3500 r/min for 10 minutes, and the supernatant was transferred to a centrifugal tube (5 mL). The sample was then extracted with CH_2_Cl_2_ (1 mL) by shaking for 4 min. and centrifuging at 13,000 r/min for 10 minutes for two times. The combined dichloromethane solution in a 10 mL sterilized centrifugal tube was evaporated by blowing nitrogen gas at room temperature, and to the residue was added 0.5 mL of the mobile phase (0.025 mol/L aqueous phosphoric acid and acetonitrile in a ratio of 83:17), after shaking for 4 min, *n*-hexane (2 mL) was added, after centrifuging for 10 min at 4 °C with 13,000 r/min speed, the aqueous solution was separated from organic solvent and filtered through 0.22 μm filter for HPLC analysis.

## Results and discussion

**Figure 2.**
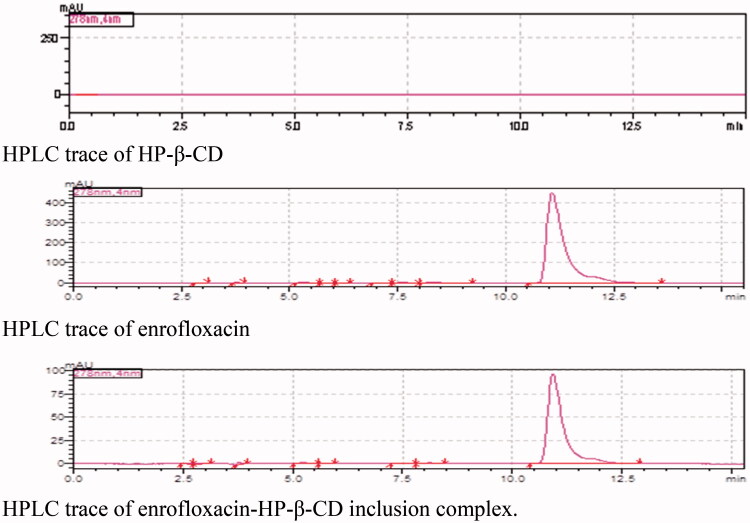
HPLC traces of HP-β-CD, enrofloxacin, and their inclusion complex.

For the inclusion complex formation, it is known that the inclusion effect of enrofloxacin is affected by the reaction temperature, stirring speed, and reaction time. HP-β-CD was put in a small amount of distilled water, and the resulting mixture was stirred with certain speed on a magnetic stirrer at specific temperature to get an HP-β-CD saturated aqueous solution. A solution of enrofloxacin in acetic acid was added slowly, and after stirring at a specified temperature for a particular time, the mixture was cooled down to room temperature and continued to stir at room temperature for a specific time, the solution was stored in a refrigerator at 4 °C for 24 hours. The solid product was obtained through the filtration. After washing with small amount of ethanol and drying at 55 °C for 24 hours, the inclusion complex was obtained as white solid. The HPLC was used to analyze the inclusion complex, the HPLC traces of HP-β-CD, enrofloxacin, and their inclusion complex are shown in Figure 2.

Accordingly, for the preparation of enrofloxacin inclusion complex, various conditions were tried, such as the ratio of enrofloxacin and HP-β-CD from 1:1 to 1:3, different stirring speeds from 400 rpm to 600 rpm, various stirring temperatures from 55 °C to 65 °C, and stirring times from 2 hours to 4 hours. More than 50 conditions were attempted to optimize the enrofloxacin inclusion complex preparation procedure. Each result was analyzed by HPLC to understand the inclusion rate and the yield of the complex. The best condition was found to be a 1:1 ratio of enrofloxacin and HP-β-CD, a stirring speed of 500 rpm, a reaction temperature of 55 °C, and a reaction time of five hours. Under these conditions, the resulted enrofloxacin inclusion complex gave the highest inclusion yield as 91.85% and the highest inclusion ratio as 91.26%.

Fourier-transform infrared spectroscopy is an excellent analytical tool for confirming the formation of the inclusion complexes. In the enrofloxacin–HP-β-CD inclusion complex, the non-covalent interaction such as hydrophobic interactions, van der Waals interactions, and hydrogen bonding between the HP-β-CD and enrofloxacin lead to the lower energy of the included part of enrofloxacin and reduce the peak intensities of the corresponding frequencies. Accordingly, if the IR absorption peaks decrease, shift or disappear, it indicates that the enrofloxacin and HP-β-CD have an inclusion effect.

**Figure 3.**
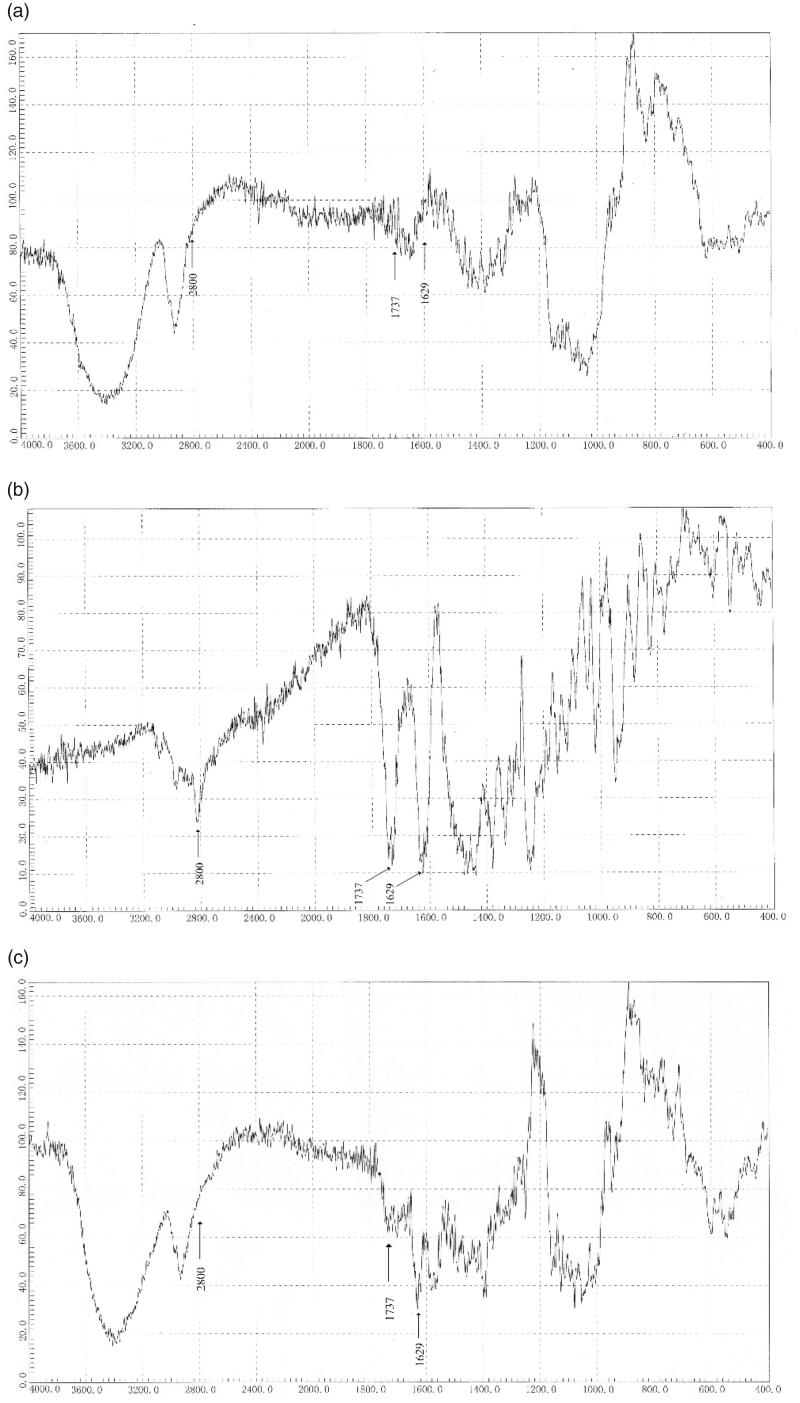
Fourier-transform infrared spectroscopy spectra of HP-β-CD (a), enrofloxacin (b), and enrofloxacin–HP-β-CD inclusion complex (c).

As shown in Figure 3, the characteristic peaks in FTIR spectrum of enrofloxacin (b) appeared at 1629 cm^−1^ (C=O stretching) and 1737 cm^−1^ (CO_2_H), through the comparison with the FTIR spectra of HP-β-CD complex (a) and the inclusion complex (c), the FTIR spectrum of enrofloxacin/HP-β-CD inclusion complex (c) showed these two peaks moved to lower frequencies (higher energy) with reduced intensities. Both keto groups of enrofloxacin were moved to higher energy frequencies in the inclusion complex is an indication of its formation of complex, since the keto groups are entrenched in the HP-β-CD cavity.

The aliphatic vibration absorption of (C–H) at 2800 cm^−1^ of enrofloxacin was disappeared after the formation of inclusion complex indicated the piperazine part of enrofloxacin was contained within the HP-β-CD cavity by van der Waals forces and hydrophobic interactions, and the C–H vibrations in enrofloxacin were affected (Figure 1).

**Figure 4.**
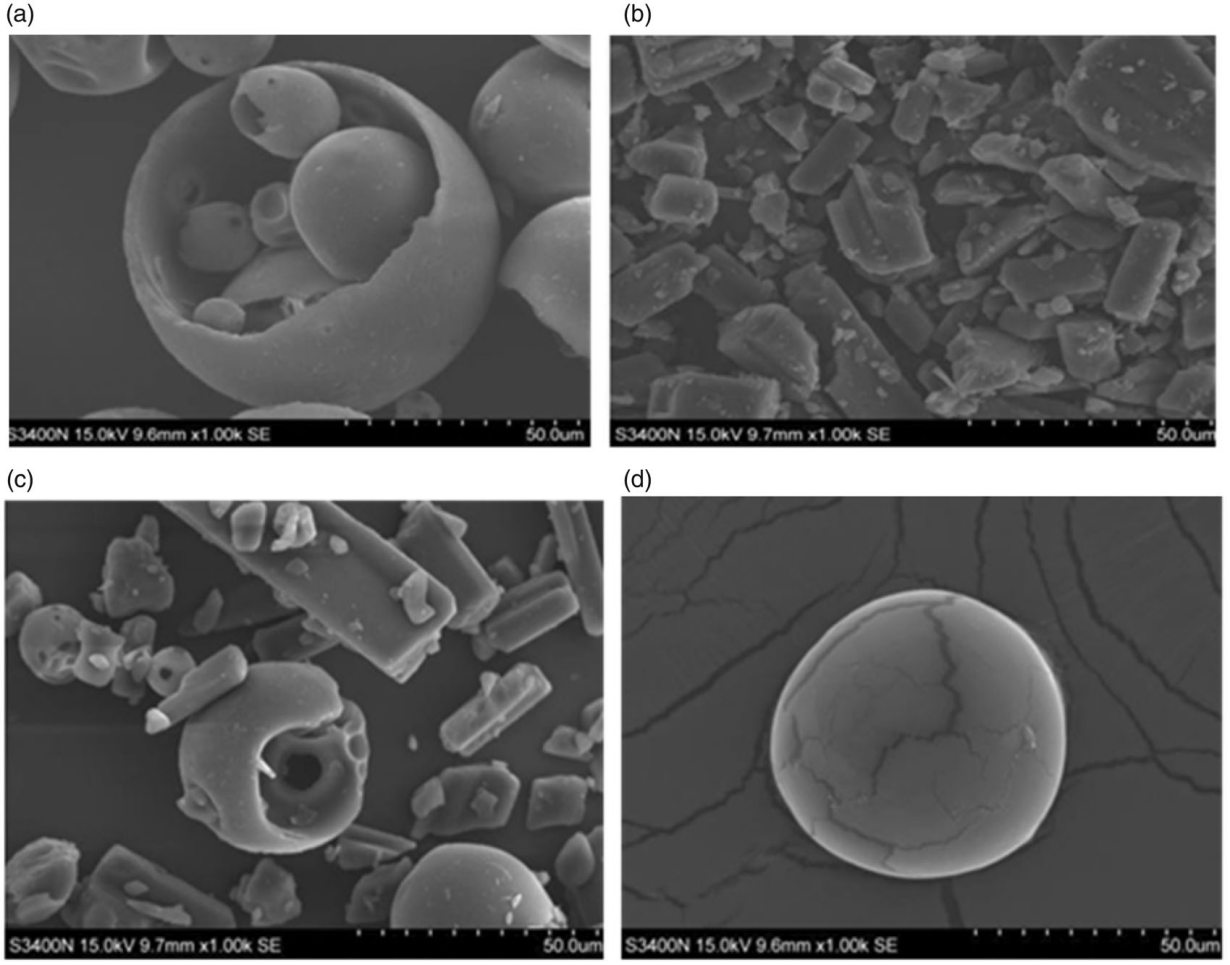
The scanning electron microscope images of the HP-β-CD (a), enrofloxacin (b), the mixture of enrofloxacin and HP-β-CD (c), and the enrofloxacin–HP-β-CD inclusion complex (d).

Whereas, the scanning electron micrographs reflect on the changes of the surface morphology of enrofloxacin and its inclusion complex. The micrographs of enrofloxacin and its inclusion complex are illustrated in Figure 4. The HP-β-CD was appeared as ball shape crystals (Figure 4(a)), the enrofloxacin in pure form appeared as lamellar crystals (Figure 4(b)), the micrographs of the mixture of enrofloxacin and HP-β-CD appeared as a mixture of ball shape crystals and lamellar crystals Figure 4(c), while the micrograph of inclusion complex displays the ball-like structures with parallelogram (Figure 4(d)) (Zheng & Chow, 2009). This change of the morphology of enrofloxacin from its inclusion complex, directly confirms the formation of the enrofloxacin–HP-β-CD inclusion complex.

**Figure 5.**
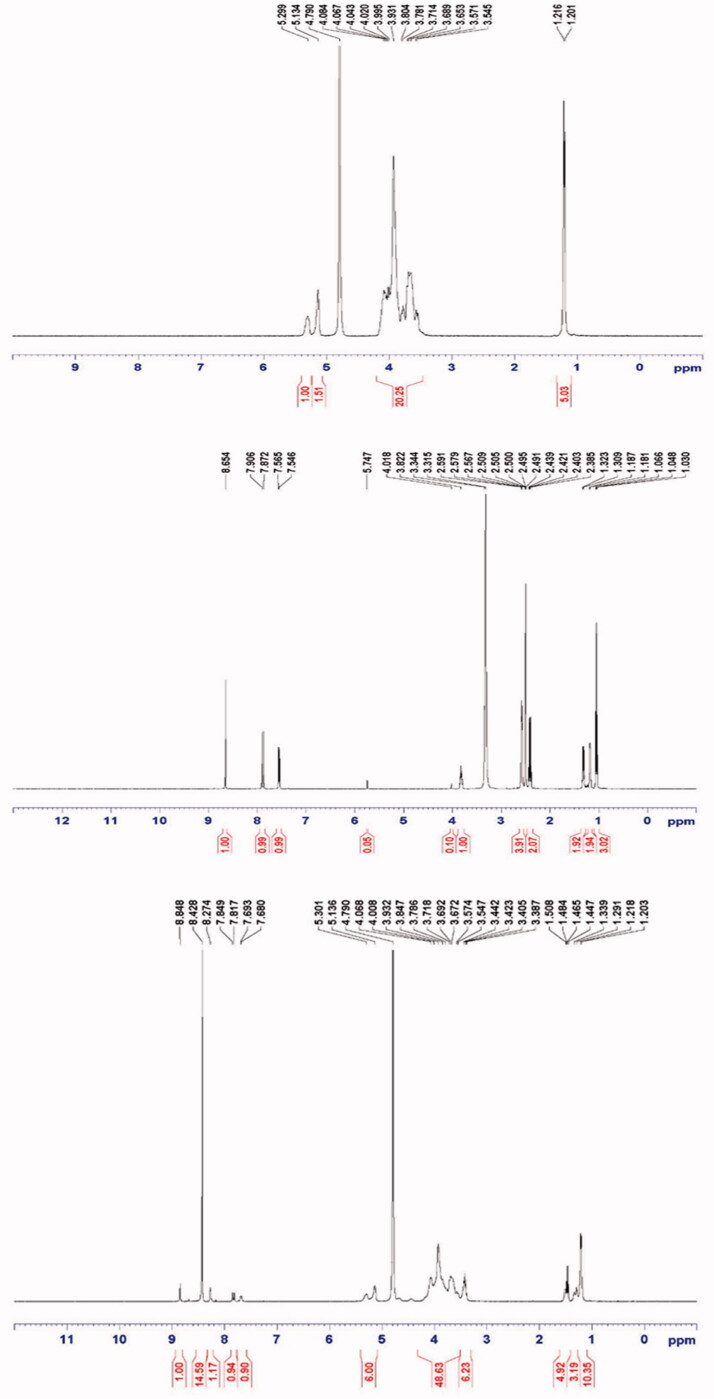
Proton NMR spectra of HP-β-CD, enrofloxacin, and their inclusion complex.

The proton NMR spectra of enrofloxacin, HP-β-CD, and their inclusion complex were recorded (Figure 5). The proton NMR spectrum of the inclusion complex exhibited the signals for enrofloxacin at 8.85 ppm (1H, s), 7.83 ppm (1H, d, *J* = 12.8 Hz), 7.68 ppm (1H, d, *J* = 5.2 Hz), 3.63–3.40 ppm (8 H, m), 1.51 ppm (2 H, d, *J* = 9.6 Hz), 1.46 ppm (3 H, t. *J* = 7.6 Hz), 1.34 ppm (2 H, d, *J* = 9.6 Hz); while the proton NMR spectrum of enrofloxacin exhibited the signals at 8.65 ppm (1H, s), 7.88 (1H, d, *J* = 13.6 Hz), 7.55 ppm (1H, d, *J* = 7.6 Hz), 3.83 ppm (1H, m), 2.59–2.38 ppm (8 H, m), 1.31 ppm (2 H, d, *J* = 5.6 Hz), 1.18 ppm (2 H, d, *J* = 5.6 Hz), and 1.05 (3 H, t, *J* = 7.2 Hz). As expected, the downward shift (higher ppm values) of chemical shift values for enrofloxacin protons in inclusion complex confirmed the formation of inclusion complex, and based on the integrations of proton NMR spectra, it is clear that the ratio of enrofloxacin and HP-β-CD in the complex is 1:1.

**Figure 6.**
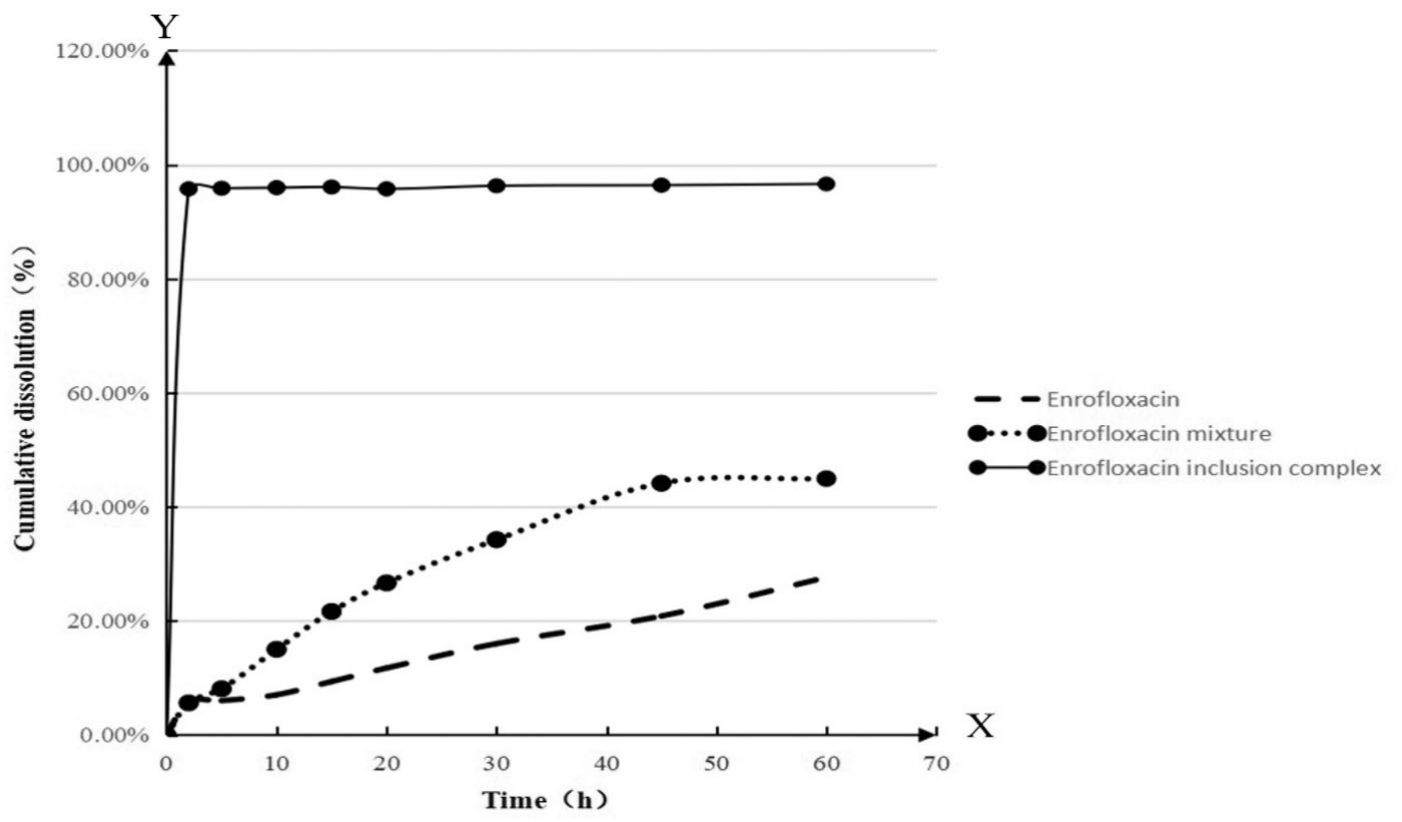
The dissolution curves of enrofloxacin, the mixture of enrofloxacin and HP-β-CD, and enrofloxacin–HP-β-CD inclusion complex.

The dissolution curves of enrofloxacin, the mixture of enrofloxacin and HP-β-CD, and enrofloxacin–HP-β-CD inclusion complex are shown in Figure 6. From these curves, it is apparent that the dissolution of enrofloxacin–HP-β-CD inclusion complex was significantly higher than that of enrofloxacin and the mixture of enrofloxacin and HP-β-CD, conclusively. At 15 min, the cumulative dissolution of the clathrate reached 100%, which was five times better than enrofloxacin alone.

The water solubility of enrofloxacin was reported as low as 0.23 mg/mL. The saturated aqueous solution of enrofloxacin–HP-β-CD inclusion complex was prepared, and its enrofloxacin content was determined by HPLC analysis. The aqueous solubility of enrofloxacin inclusion complex is found to be 210 mg/mL, which is 916-folds higher than that of enrofloxacin alone.

The stability of the enrofloxacin–HP-β-CD inclusion complex upon storage was also examined. The inclusion complex was stored at 4 °C in a sealed tube in a refrigerator for six months, and its HPLC analysis showed no changes in the enrofloxacin’s content.

**Figure 7.**
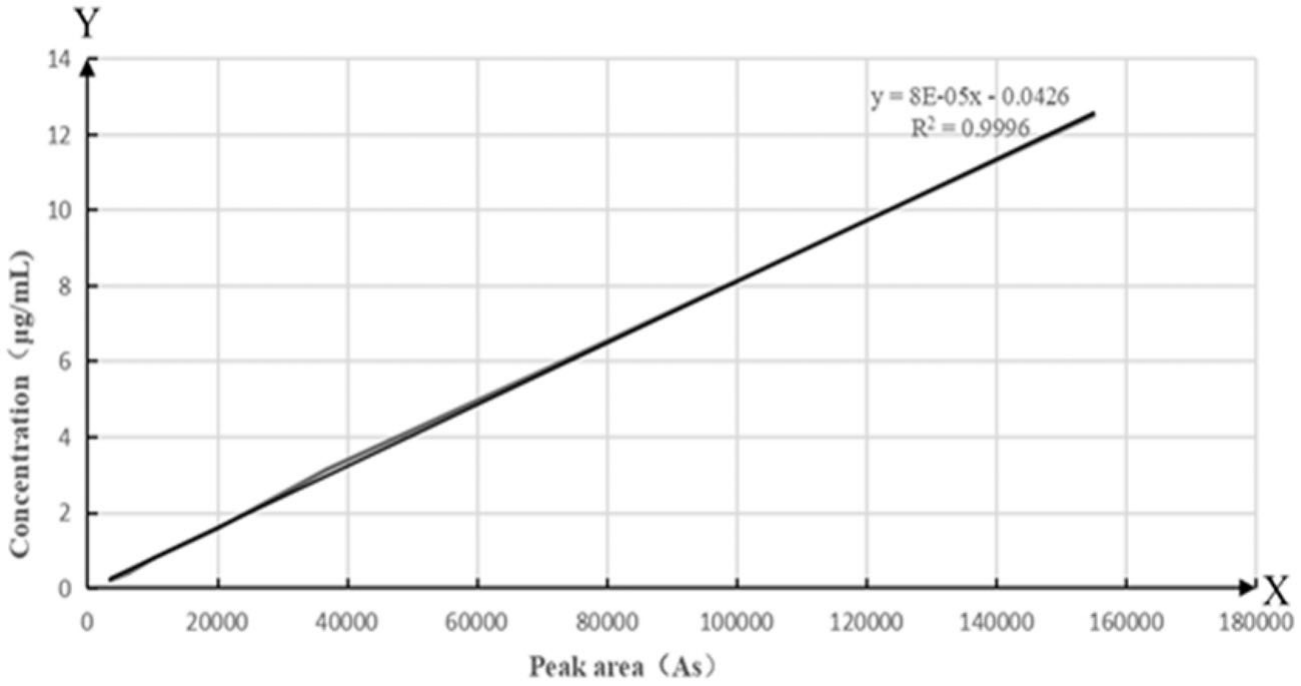
The standard curve of enrofloxacin in rat blank plasma.

The pharmacokinetic properties of a drug are influenced when they are complexed with a carrier. To understand the influence of HP-β-CD complexation of enrofloxacin, a pharmacokinetic study was designed. Accordingly, enrofloxacin and its HP-β-CD inclusion complex (based on the effective content of enrofloxacin) were orally dosed to SPF rats in two groups at a single dose of 5 mg/kg of body weight, and the blood samples were collected at 0.25, 0.5, 0.75, 1, 2, 3, 4, 6, 8, 12, 24, 36, 48, and 72 hours right after drug administration, respectively. The standard working curve is shown in Figure 7, which was obtained from the HPLC analysis of rat blank plasma and rat plasma with enrofloxacin. When the concentration of enrofloxacin in plasma was ranged from 0.2 to 12 µg/mL, the peak area in HPLC and concentration showed linear relationship, and the linear regression equation and correlation coefficient are obtained as *Y* = 8^−5^*X*–0.0426 (*R*^2^=0.9996; *Y*: peak area; *X*: concentration), LOQ was 0.2 µg/mL, and LOD was 0.1 µg/mL.

**Figure 8.**
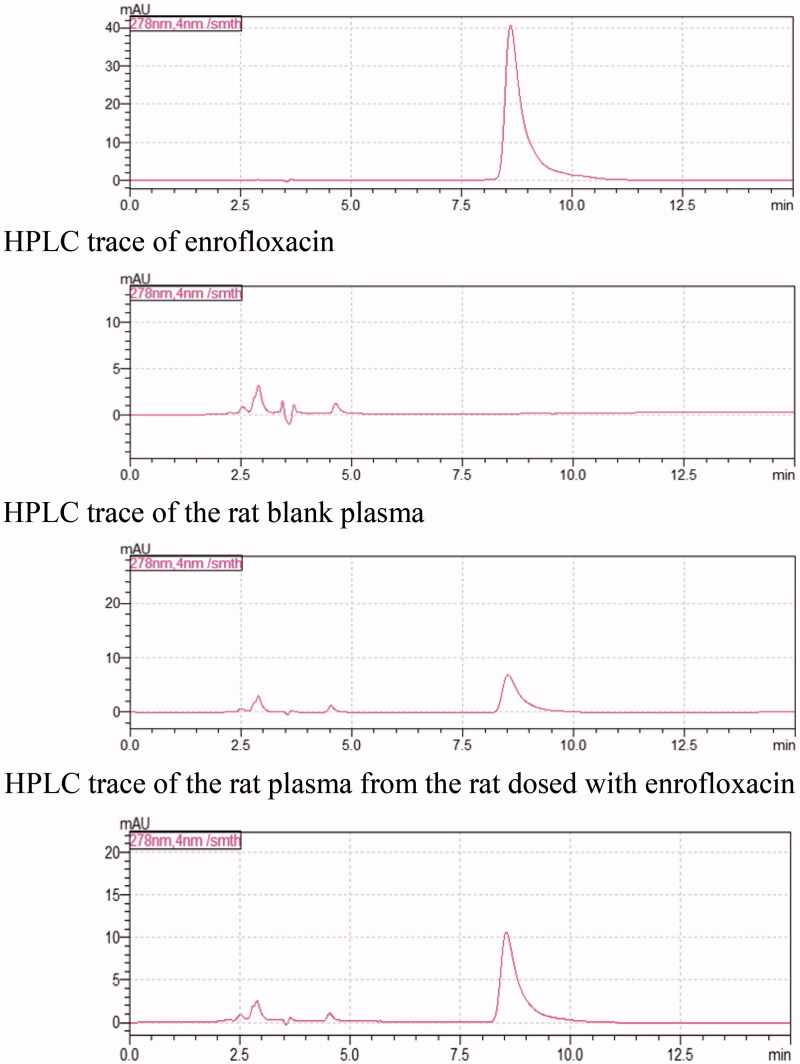
HPLC traces of enrofloxacin, rat blank plasma, and plasma samples from the rats dosed with enrofloxacin or its HP-β-CD inclusion complex.

The HPLC traces of pure enrofloxacin, the rat blank plasma, the rat plasma from the rats dosed with enrofloxacin, and the rat plasma from the rats dosed with enrofloxacin inclusion complex are displayed in Figure 8.

**Figure 9.**
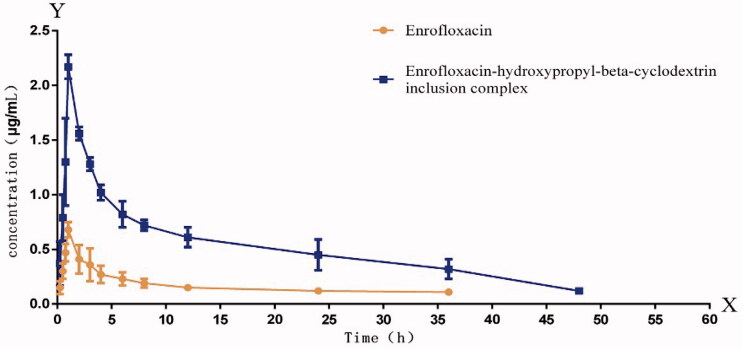
Curve of drug concentrations in plasmas from rats dosed with enrofloxacin or its inclusion complex.

The above HPLC traces clearly indicated that the rat blank plasma did not interfere with the detection of enrofloxacin, and these HPLC conditions can be used to determine the concentrations of enrofloxacin in the rat plasma samples. The collected plasma samples were analyzed by HPLC, and the results are shown in Figure 9.

Based on the data (by using WinNonlin 5.2.1), it is concluded that the pharmacokinetic parameters of enrofloxacin and its HP-β-CD inclusion complex in healthy rats conformed to the first-order absorption two-compartment model. The pharmacokinetic parameters (mean ± SD) are presented in Table 1.

**Table 1.** Pharmacokinetic parameters of enrofloxacin and its inclusion complex.

Parameters	Unit	Value	
		Enrofloxacin	Enrofloxacin–HP-*β*-CD
*T*_max_	h	1.53	1.92
*C*_max_	μg/mL	0.46	1.40
AUC	μg·h/mL	12.50	25.97
*t*_1/2_	h	7.98	5.91
CL	L/kg/h	400.15	192.56

After oral administration, *C*_max_ of enrofloxacin and enrofloxacin/HP-β-CD conclusion complex in plasma of rats were found to be 0.46 µg/mL (at 1.53 h) and 1.40 μg/mL (at 1.92 h), respectively. Interestingly, in the enrofloxacin–HP-β-CD group, *C*_max_ (1.40 μg/mL) was considerably higher than that in the enrofloxacin group (*C*_max_: 0.46 μg/mL). While the AUC_0–∞_ of HP-β-CD conclusion complex was determined as 25.97 μg h/mL which was 2.08-folds higher than that of enrofloxacin (12.50 μg h/mL) alone. Further rate of clearance of enrofloxacin–HP-β-CD (192.56 L/Kg/h) is halved by that of enrofloxacin alone (400.15 L/kg/h).

In general, the inclusion complexes have a sustained release effect on the drug release, especially in the later stages, as drug release is slower than in initial stages and the amount of drug is reduced, and HP-*β*-CD molecules are relatively abundant. It can be seen from the table, that the differences in *C*_max_, *T*_max_, AUC_0–∞_, and *t*_1/2_ between enrofloxacin and enrofloxacin–HP-*β*-CD conclusion complex were significant. The increased water solubility (916-fold higher) of the drug in complexation with HP-*β*-CD might be responsible for the resultant increased values of *C*_max_ and AUC_0–∞_ of enrofloxacin/HP-β-CD conclusion complex than those of the free enrofloxacin. Theabove data clearly indicate a better absorption of enrofloxacin, entrapped in HP-β-CD inclusion complex, in the gastrointestinal tract. Thus, the pharmaceutical properties of absorption and bioavailability of enrofloxacin have been significantly improved by complexation with HP-β-CD.

## Conclusions

In conclusion, we have devised a reliable and consistent method to prepare an inclusion complex of enrofloxacin–HP-β-CD with an improved aqueous solubility (916-fold) in comparison to that of enrofloxacin itself. The inclusion complex has been characterized by FTIR, ^1^H NMR, and SEM techniques. As originally hypothesized, the pharmacokinetic evaluation of inclusion complex of enrofloxacin/HP-β-CD in rats showed that the pharmaceutical properties of absorption (*C*_max_: 0.46 vs. 1.40 μg/mL) and bioavailability (12.5 vs. 25.97 μg·h/mL) of enrofloxacin were significantly improved.
